# Mechanisms for Mid-Air Reorientation Using Tail Rotation in Gliding Geckos

**DOI:** 10.1093/icb/icab132

**Published:** 2021-06-18

**Authors:** Robert Siddall, Victor Ibanez, Greg Byrnes, Robert J Full, Ardian Jusufi

**Affiliations:** Locomotion in Biorobotic and Somatic Systems Group, Max Planck Institute for Intelligent Systems, Heisenbergstraße 3, 70569 Stuttgart, Germany; Locomotion in Biorobotic and Somatic Systems Group, Max Planck Institute for Intelligent Systems, Heisenbergstraße 3, 70569 Stuttgart, Germany; Neuroscience Center, University of Zurich, Winterthurer Strasse 190, 8057 Zürich, Switzerland; Department of Biology, Siena College, 515 Loudon Road, Loudonville, NY 12211, USA; Department of Integrative Biology, University of California, Berkeley, 3040 Valley Life Sciences Building 3140, Berkeley, CA 94720-3140, USA; Locomotion in Biorobotic and Somatic Systems Group, Max Planck Institute for Intelligent Systems, Heisenbergstraße 3, 70569 Stuttgart, Germany

## Abstract

Arboreal animals face numerous challenges when negotiating complex three-dimensional terrain. Directed aerial descent or gliding flight allows for rapid traversal of arboreal environments, but presents control challenges. Some animals, such as birds or gliding squirrels, have specialized structures to modulate aerodynamic forces while airborne. However, many arboreal animals do not possess these specializations but still control posture and orientation in mid-air. One of the largest inertial segments in lizards is their tail. Inertial reorientation can be used to attain postures appropriate for controlled aerial descent. Here, we discuss the role of tail inertia in a range of mid-air reorientation behaviors using experimental data from geckos in combination with mathematical and robotic models. Geckos can self-right in mid-air by tail rotation alone. Equilibrium glide behavior of geckos in a vertical wind tunnel show that they can steer toward a visual stimulus by using rapid, circular tail rotations to control pitch and yaw. Multiple coordinated tail responses appear to be required for the most effective terminal velocity gliding. A mathematical model allows us to explore the relationship between morphology and the capacity for inertial reorientation by conducting sensitivity analyses, and testing control approaches. Robotic models further define the limits of performance and generate new control hypotheses. Such comparative analysis allows predictions about the diversity of performance across lizard morphologies, relative limb proportions, and provides insights into the evolution of aerial behaviors.

## Introduction

Locomotor performance can influence success in nearly all facets of an organism’s life, including avoiding predation, and searching for food or suitable mates (Turchin 1998; Nathan et al. 2008). Arboreal habitats present numerous challenges to locomotor performance due to unsure footing on steep or narrow and often discontinuous substrates. These challenges are made all the more daunting considering animals are often moving on these substrates tens of meters above the ground below. Any mechanism that can provide an additional point of contact with the substrate or to maintain balance in case of the slip of a foot, could prevent a fall or if a fall does occur, the ability to control body posture to land safely can be the difference between life and death. Many organisms use their tails to accomplish these tasks by either increasing contact with the substrate, or by using inertial or aerodynamic forces to aid in postural control.

Numerous primates (German 1982; Hunt et al. 1996; Schmitt et al. 2005) and other mammals (McClearn 1992) are thought to use tails to provide an additional point of contact while reaching (McClearn 1992), climbing or moving on narrow substrates (Cunha and Vieira 2002; Lemelin and Cartmill 2010; Dalloz et al. 2012), or crossing gaps between branches (Graham and Socha 2020). Some reptiles including snakes (Byrnes and Jayne 2012) and chameleons (Herrel et al. 2011) also grip with their tails during gap-crossing. Many other animals including birds (Norberg 1986; Fujita et al. 2007, 2008) and mammals (McEvoy 1982) are thought to use their tail as a prop while climbing steep substrates. The most extreme example of this behavior is the dynamic “kickstand” response elicited by forefoot slippage in rapidly climbing geckos (Jusufi et al. 2008) in which the tail is pressed against the substrate in response to loss of contact between the foot and surface to arrest pitch-back and avoid falling from the vertical surface being climbed. Overall, most previous observations have focused on modes of locomotion that are not rapid, or characterized steady state behavior without perturbations.

In addition to these mechanisms to increase contact with the substrate, tails and other appendages can be used to reorient the body using inertia while moving across a surface or while airborne. Cursorial mammals, such as cheetahs (Wilson et al. 2013), and lizards (; Clemente and Wu 2018) use their tails to change body orientation while running over variable terrain. Tails are also used to change body orientation in mid-air by leaping (Dunbar 1988, 1994) or saltatory (Freymiller et al. 2019) animals. The risks of falling have shaped behavior in a wide variety of arboreal taxa resulting in reorientation after becoming airborne (Jusufi et al. 2010; Jusufi et al. 2011) to minimize the risk of injury or control the flight path (Dudley et al. 2007). Gliding behaviors appear to evolve at the same time as the development of rainforests (Heinicke et al. 2012), and the subject of the paper (*Hemidactylus platyurus*) is known to exhibit “parachuting” locomotion. Despite not possessing the more developed aerodynamic adaptations of other gecko species (Young et al. 2002) *H. platyurus* still exhibits a dorsoventrally flattened body and tail. Aerial-righting behaviors have been described in a wide range of taxa from insects (Jusufi et al. 2011; Ribak et al. 2013; Munk et al. 2015; Zeng et al. 2017) to lizards (Jusufi et al. 2010) to cats (Marey 1894; Kane and Scher 1969) and often use tail inertia to aid in reorientation while falling.

During glides, tails have also been implicated in the volitional maneuvers of many taxa, both living (e.g., Thorington and Heaney 1981; Dudley and Yanoviak 2011; Socha et al. 2015) and extinct (e.g., Stein et al. 2008; Evangelista et al. 2014). These mechanisms could rely on appendage inertia (Jusufi et al. 2008; Jusufi et al. 2010) or aerodynamic forces acting on an outstretched appendage (e.g., McCay 2001; Zeng et al. 2017). In reptiles, numerous anecdotal descriptions have been made of tail use in maneuvering flight (e.g., Colbert 1967; Young et al. 2002), but little quantitative data exist on the mechanics of tail use during maneuvers in gliders, especially the independent contributions of inertia and aerodynamic forces. Quantitative studies of tail rotational inertia in geckos have shown that tail inertia can be used to change pitch and yaw during gliding (Jusufi et al. 2008) using similar movements as during aerial righting (Jusufi et al. 2008, 2010, 2011). By comparing lizard species with different tail proportions it was shown that longer tail lengths can reduce the tail motion required to elicit maneuvers of similar magnitude (Jusufi et al. 2010, 2011).

In cases where animal performance data are limited, it is possible to use robotic modeling to inform the mechanical basis of locomotor behavior (Roderick et al. 2017). Wind tunnel testing of models is widely used to inform aerodynamics (McCay 2001), and recently the development of precise miniaturized mechatronic components have allowed testing of active robotic models in free flight conditions, allowing access to a wider envelope of motion, that includes free rotation relative to the free stream (as opposed to the prescribed body orientations used in a statically mounted wind tunnel test), incorporating dynamic as well as static effects.

Many robotic models testing the uses of tails in locomotion have been presented recently. Inertial tails have been used for robot steering on the ground (Pullin et al. 2012; Kohut et al. 2012; Patel and Braae 2013), and in aerial righting. Tail inertia has also been used to control roll orientation during free-fall (Jusufi et al. 2010) and pitch orientation during jumping (Johnson et al. 2012; Libby et al. 2012; Yim and Fearing 2018), and to passively absorb perturbations in terrestrial locomotion (Siddall et al. 2021). The combination of aerodynamic and inertial control has also been employed to yaw a robot (Kohut et al. 2013).

To understand the role of tail mechanics in pitch and yaw control and the independent effects of both rotational inertia and aerodynamic forces on reorientation during gliding in lizards, a combination of animal data and mathematical and robotics models were used. Kinematic data from the Asian Flat-Tailed Gecko (*H. platyurus*) were examined to determine what tail movements result in reorientation of the body in pitch and yaw. Based on these data, a mathematical model was developed to describe the effect of tail length on turning performance. Finally, a robotic model was used to determine the independent effects of tail inertia and aerodynamic forces on body reorientation in pitch during gliding.

## Materials and methods

### Animal experiments in a wind tunnel

**Fig. 1:**
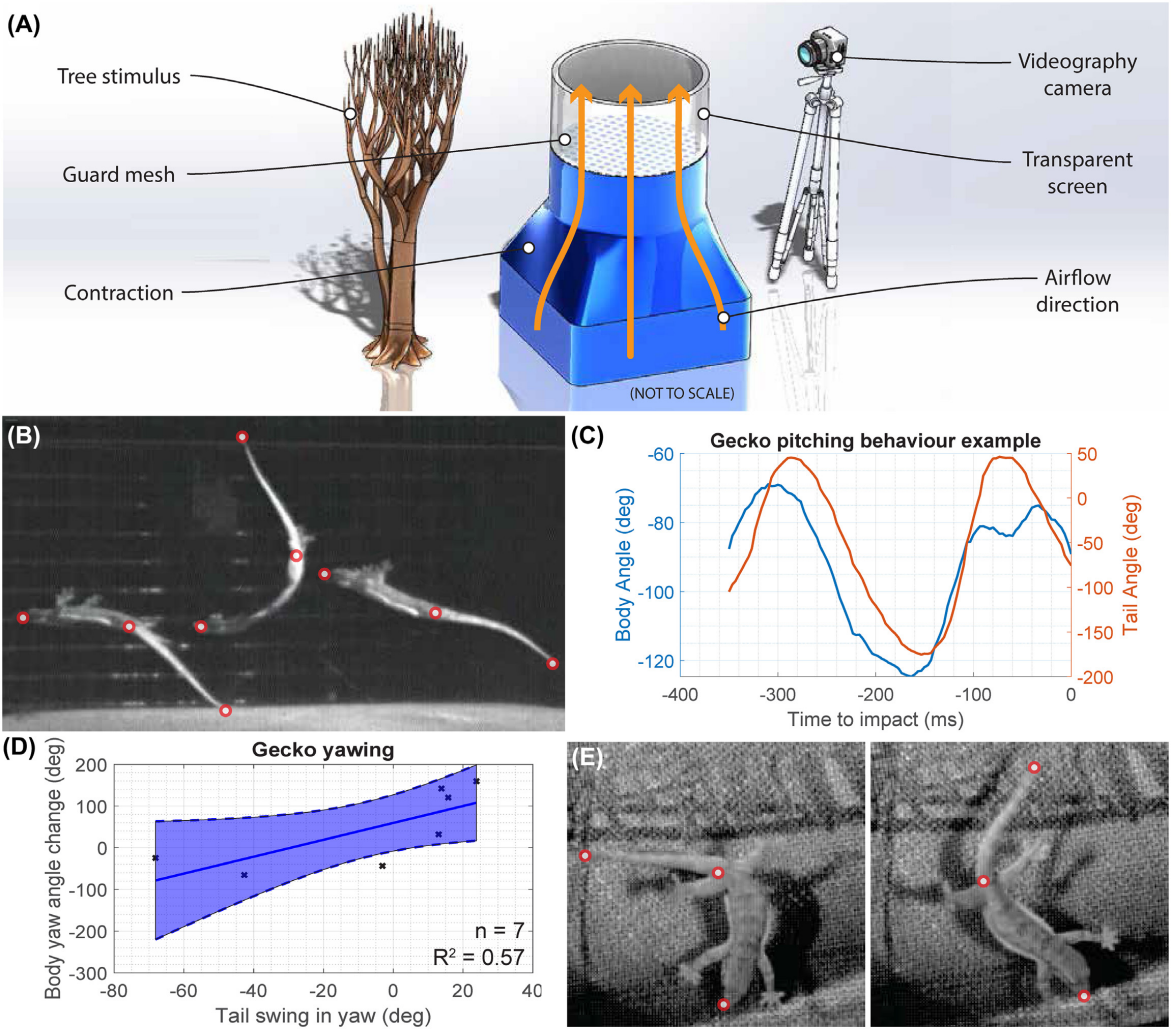
Wind tunnel gliding experiments with geckos *H. platyurus*. (**A**) Illustration of the experimental set up, showing layout of key components, not to scale. (**B**) Composite image of a gecko in sagittal view performing ventral and dorsal flexion of its tail in flight to alter body pitch, with tracked locations indicated. Footage from Jusufi et al. 2008, sequence 6, modified. (**C**) Tracked body and tail angles during tail motion in pitch, showing body motion following tail motion. (**D**) Tail motion against body motion in yaw, collecting seven tracked tail swings, and showing correlation between tail and body motion. (**E**) Example trial of gecko in dorsal view with tail yaw rotation, showing start and end point, with tracked locations indicated.

In this paper, we expand upon an experimental set up previously described in Jusufi et al. (2008, 2010), presenting tracked kinematics of separate data sets acquired through high-speed videography and testing the cross-correlation of tail and body motion. The gliding behaviors of geckos were investigated with an open circuit vertical wind tunnel, with a working section flow speed of 2–8 m/s. The animals were placed in the transparent tunnel working section, above the contraction and fan, with Plexiglas and mesh screens used to prevent animals from leaving the tunnel or entering the contraction. A tree stimulus was placed within sight of the working section, as a target for the animals, and cameras (AOS X-pri) were placed outside the tunnel to record motion (Fig. 1A). Anemometers (VelociCalc; TSI, Inc.) were used to measure airspeed, and geckos were found to glide at speeds between 4 and 7 m/s, depending on the mass and surface area of the individual.

We analyzed four gecko “flights” across the vertical wind tunnel cross-section (three recorded from above, tracking yaw, and one recorded from the side, tracking pitch), marking body and tail motion. Total gecko body length including tail was 9.86 ± 0.08 mm, and animal mass was 2.85 ± 0.3 g. Videos were recorded at 205–300 fps, and were marked manually using DLTdv8 (Hedrick 2008), tracking snout, vent and tail tip locations. Data were post processed in Matlab to extract body and tail angles. Body angle was defined as the angle of a straight line drawn from the gecko snout to vent marker, relative to the camera coordinate system/inertial reference frame. Tail angle was calculated as the relative angle between a straight line drawn from tail tip to vent and the line drawn between snout and vent, with a tail angle of zero representing the tail being parallel with the body (i.e., snout, vent and tail tip lying along one line). We observed geckos swinging their tails in both pitch and yaw while trying to reach a tree stimulus placed in sight of the tunnel. Tail motions in both pitch and yaw were observed to match with opposing motion of the body, as expected based on angular momentum conservation; Fig. 1B–E). All experimental procedures were approved by The Animal Care and Use Committee at University of California, Berkeley.

### Multibody model of tail motion

**Fig. 2:**
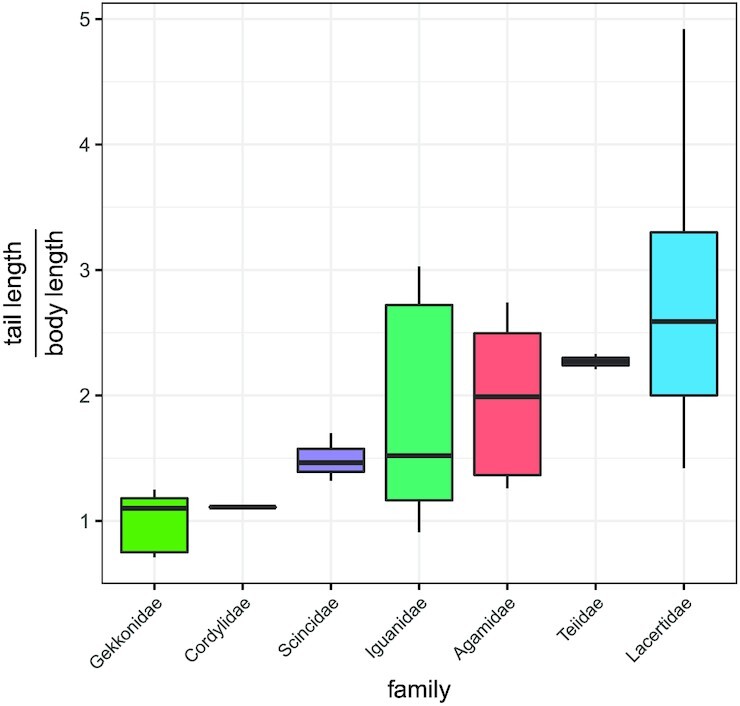
Examples of relative tail size variation across several lizard families (n = 33). Body length was measured snout to vent, tail length was measured from vent to distal tail tip. Gekkonidae (H. platyurus) tend to have relatively short tails compared to body length (approx. 1:1). Dimensions for *Agamidae* were taken from preserved specimens at the Harvard Museum of Comparative Zoology. For all others they are processed from Tail autotomy, tail size, and locomotor performance in lizards. McElroy, Eric J and Bergmann, Philip J. Physiological and Biochemical Zoology.

While *H. platyurus* tails are typically around the same length as their body, many other lizard families exhibit much larger tail lengths (Fig. 2). To gain more insight into the mechanics of inertial reorientation with tails, a simulation was developed in Matlab/Simulink, using the Simscape Multibody package. The simulation models the animal’s tail and body as two rigid bodies, connected by two pin joints in the roll and yaw axes. The body and tail are all modeled as rigid bodies of uniform density, with the torso represented as a rectangular prism, the legs as cylinders and the tail as a cone. Dimensions and masses for *H. platyurus* are taken from Jusufi et al. (2010).

**Fig. 3:**
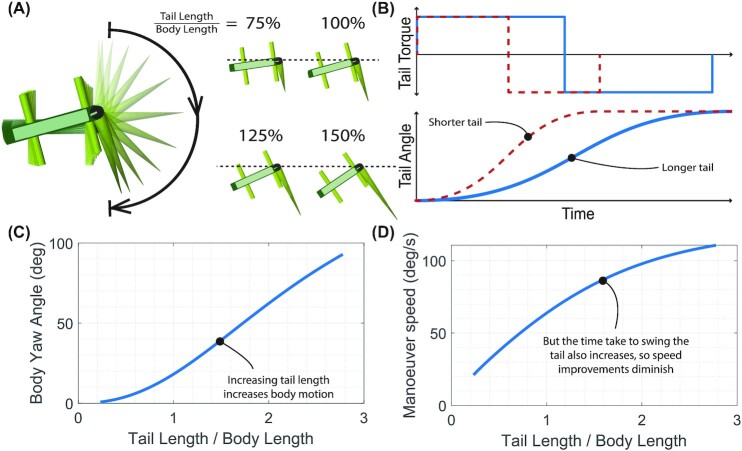
Simulating a planar yaw over a range of tail lengths. (**A**) Graphical output from the simulations, showing a tail motion sequence, and the final position after a tail swing for four different tail lengths. (**B**) Illustrative plot of how actuation changes as tail length is increased—accelerating and decelerating torque is kept constant, but actuation time changes. (**C**) Body angle change after 180° tail swing. (**D**) Maneuver speed shows diminishing returns, as the actuation time increases more rapidly than total body movement.

Initially, we modeled a simple planar 180° swing of the tail in yaw, in a motion similar to the maneuvers observed in the wind tunnel testing (Fig. 1E), and used the model to test the effect of tail length (Fig. 3A). To produce the swing in the simplest possible manner, we applied a constant torque to the tail for 90° of swing, followed by an equal decelerating torque for the next 90°, such that the tail came to rest at 180° (Fig. 3B). The tail was then scaled up, while keeping tail base diameter and material density constant. The resulting body motion was analyzed in terms of both the angle moved (Fig. 3C) and the time taken for the swing (Fig. 3D). As tail length was scaled up, we held actuator torque constant, reasoning that the available muscular torque to accelerate the tail would scale with tail thickness rather than length.

### Tail motion in multiple degrees of freedom

**Fig. 4:**
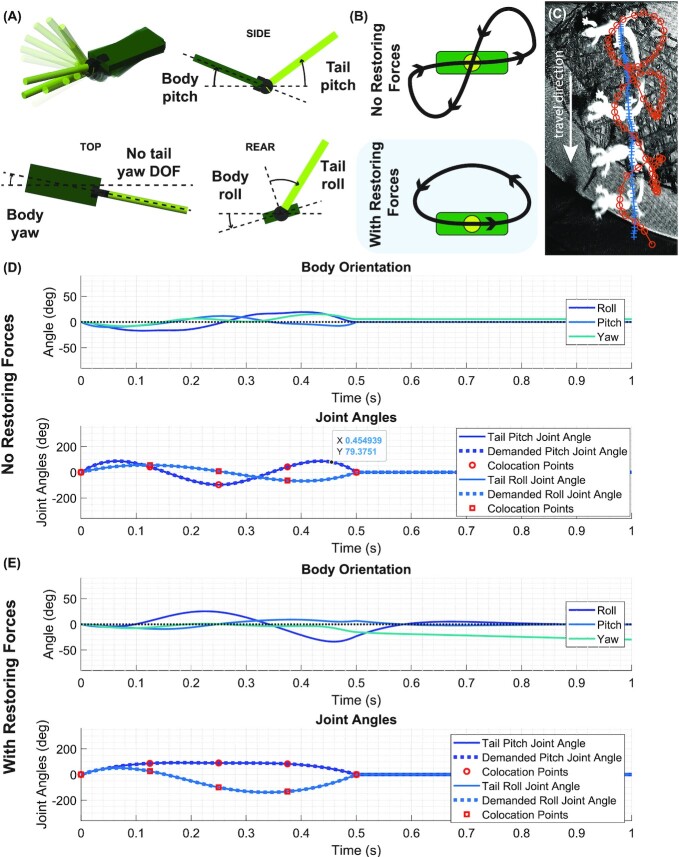
Tail motion in multiple degrees of freedom, produced via trajectory optimization in a simulated tail. (**A**) Graphical simulation output, showing axis definitions. (**B**) Without external forces, trajectory optimization produces “figure eight” motion, that does not resemble nature, but with external forces, tail motion resembles gecko observations. (**C**) The cyclic tail swings that produce a yawing of the body in the gliding gecko. Lines show the position of the tail base (blue “+”) and the tail tip (red “o”). (**D**) without restoring forces, the tail must repeatedly change directions on the way to a 30° yaw. (**E**) with restoring forces on the pitch and roll axes, a simple tail swing is enough to produce a 30° yaw, and tail motion resembles nature.

Previous work on lizard tails has focused on tail motion with a single degree of freedom Jusufi et al. (2010). However, a planar yaw or pitch swing eventually requires some return movement in the opposite direction, in order for the tail to be used repeatedly (Patel et al. 2016). And because inertial forces are conservative, this will produce the opposite body motion to the initial movement. In practice, tail motion for inertial reorientation needs to be cyclic, with the tail returning to its neutral position (Fig. 4A). However, even a two degree of freedom tail with limited motion ranges presents an impractically large range of possible tail trajectories that produce useful body motion. The multibody gecko model provides an opportunity to apply trajectory optimization techniques to the tail, and attempt to “reverse engineer” the motions observed in nature (Shield et al. 2021).

As our animal experiments indicated both an inertial and an aerodynamic role for the tail, we also used the model to investigate the effect of external forces on inertial reorientation. While an accurate aerodynamic model of the gecko would require significant computational complexity, beyond the scope of this paper, we represented aerodynamic forces in simplified form, as a pair of linear torsion springs acting on the roll and pitch axes of the body, such that the body is at equilibrium with a pitch and roll angle of zero, while the yaw axis of the body is unconstrained. This was felt to be a reasonable abstraction of small perturbation around an equilibrium gliding posture at terminal velocity, in which only pitch and roll rotations produce movement into the airflow direction. The springs added to each axis represent a simplified version of the aerodynamic restoring torques that keep a stably gliding object at an equilibrium orientation. Our approximation ignores any effect from tail position on the aerodynamic torque, and is only dependent on body motion. Including these simple external forces was enough to modify the tail trajectory found by a trajectory search (Fig. 4B) in a way that made the simulated motion better reflect the motion observed in gliding geckos (Fig. 4C).

In this paper we have set up an optimization of the tail trajectory using a genetic algorithm to search the space of possible trajectories. To bound the dimensionality of the problem, we have parameterized the trajectory with six collocation points (three in pitch and three in roll; Fig. 4D), equally spaced in time, and defined the trajectory of the tail as a cubic spline through the points (Kelly 2017). These six points are used as input to the search algorithm, with each point constrained to be between −180° and +180°. The tail is constrained to start and finish at the same position, parallel to the body. This ensures that the resulting motion is cyclic, and can be repeated. The collocation points were spread over a 0.5 s timespan, to reflect the movement speed of the tail motions observed in the wind tunnel (Fig. 1C).

The genetic algorithm (adapted from The MathWorks Student Competitions Team 2020) conducts a stochastic search of the space of possible trajectories according to the value of an objective function (see Appendix 1 for details, including a link to a code repository). This objective function was calculated using the body pose at the end of the simulation (at 1 s simulation time). The trajectory optimization is run with a population size of 20 for 20 generations, requiring 400 simulations of the model, each with a different set of collocation points. An extended description of the model and parameter list can be found in Appendix 1.

We ran the optimization routine twice, once with no external forces on the pitch and roll axes (Fig. 3B), and once with external forces active (representing tail motion with and without aerodynamic reaction forces; Fig. 4D). The external forces act only on the body roll and pitch axes, such that an external torque acts on the body:

(1) $$\begin{equation*}
T_{\alpha } = C_1\alpha + C_2\dot{\alpha }\\
\nonumber
\end{equation*}$$

(2) $$\begin{equation*}
\nonumber\\
T_{\beta } = C_1\beta + C_2\dot{\beta }
\end{equation*}$$

where *T*_α_ and *T*_β_ are the torques acting on the pitch and roll axis, respectively, and *C*_1_ and *C*_2_ are constants. No other external forces act on the body. While a coarse representation, the inclusion of external torques can approximate oscillations about an aerodynamic equilibrium point. Many natural modes of flying systems are well approximated by damped harmonic oscillation (McCay 2001) (e.g., the short-period pitch oscillation), and the representation is enough to illustrate the effect that aerodynamic torques could potentially have on the motion of a gliding animal with an inertial tail.

### Biorobotic gliding experiments

**Fig. 5:**
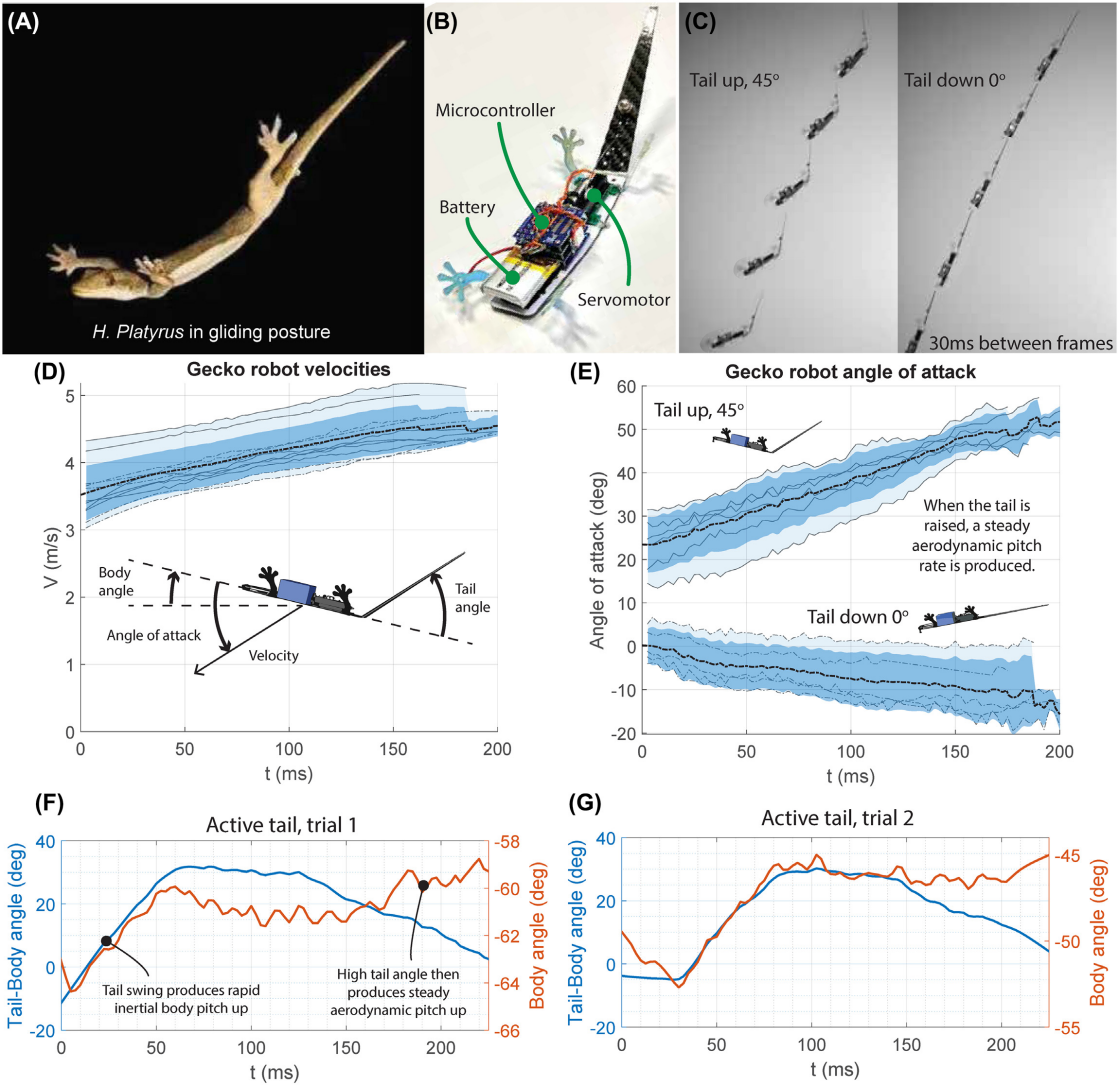
Biorobotic active tail experiments. (**A**) Image of *H. platyurus*’ gliding posture, Copyright (2008) National Academy of Sciences (Jusufi et al. 2008). (**B**) Close up view of the robot, showing the tail servo. (**C**) Image sequence from two glides with tail raised and lowered. (**D**) Descent velocity across runs. No statistically significant changes in velocity resulted from changes in tail position. (**E**) Angle of attack with different tail positions—a raised tail produces a positive pitch up, while a flat tail tends to follow the robot’s direction of travel. (**F, G**) Example runs with an active tail motion mid-flight. In both cases, the initial upward swing of the tail produces a simultaneous pitch of the body. Once the tail is raised, the body continues to pitch upward slowly from the aerodynamic torque.

Based on the importance of aerodynamics indicated by the modeling and analysis, we sought to quantify the aerodynamic effect of the tail by conducting a gliding test of a simple biorobotic model with an active tail. We built a small robot modeled on the posture of *H. platyurus* during gliding (Fig. 5A–C). We tested pitch motion of the tail, rather than yaw, as this kept the robot tail motion in the same plane as the glide motion.

Our robot has a body length of 80 mm, and a mass of 12 g. The body of the robot is made from 0.25 mm thick carbon fiber sheet, with a small nylon hinge to create a tail joint. The carbon fiber is hand cut with scissors and a dremel multitool. The arms and legs of the gecko were laser cut from 1 mm polypropylene sheet, and bent upward with a heat gun to mimic the gecko’s “skydive” posture, which is presumed to have a relevant positive influence on roll stability via the dihedral effect. The tail was actuated using a small linear servo (Spektrum SPMSH2040L), chosen because the leadscrew mechanism would not backdrive, unlike more common spur gear servos. Spring steel wire and kevlar twine are used to connect the servo output to the tail. The robot uses a SAMD21 microcontroller from TinyCircuits (ASM2021-R) powered by a LiPo battery, (150mAh, TY502020). The robot also includes an SD card reader (ASD2201-R) that is not used in this paper, but contributes to the robot’s weight (Fig. 5B).

For the glide tests, the robot was dropped and allowed to descend freely from a height of 3 m. An 0.5 m aluminum channel was used as a launch rail, to ensure consistent orientation at the start of the glide. Tail movement mid-glide was done open loop, occurring at a fixed time after release. The lower section of the robot glide was filmed with a high-speed camera (AOS S-motion) at 500 Hz (Fig. 5C), and videos were tracked using DeepLabCut (Mathis et al. 2018), with pixel calibration based on the length of the robot body, such that the length of a pixel was set by averaging the length of the body in pixels over each run to give a calibration length.

## Results and discussion

Using a combination of animal data with mathematical and robotic models, we have shown that lizards can reorient in mid-air using both inertial and aerodynamic forces from their large tails. Tail motions were associated with reorientation of the body in both pitch and yaw. From these data, two major patterns emerged. First, over the range of tails observed in most arboreal lizards, increasing tail length relative to body length increased the rate of body reorientation (Fig. 3). Second, while the lizards use both inertial and aerodynamic forces to reorient, inertial forces result in more rapid reorientation than do aerodynamic forces (Fig. 5).

### Animal data

In one glide across the wind tunnel, repeated tail motions were observed in pitch (Fig. 1B, C). Cross-correlation analysis of body and tail angle showed that tail motion slightly lagged body motion, with a peak cross-correlation at 16 ms time lag. The same was observed in another run with three repeated swings of the tail in yaw, with a time lag in this case of 24 ms. The time lag indicates the presence of an aerodynamic effect. If the body motion was due entirely to inertial effects, we would expect peak cross-correlation at zero time lag, because the motion derives from conservation of momentum, meaning instantaneous body angular velocity would be correlated with instantaneous tail angular rate. On the other hand, aerodynamic torques and the resulting angular accelerations are principally proportional to tail position (a large tail deflection typically presents a larger area to the oncoming flow, and so produces more aerodynamic force), such that the change in body angular velocity from aerodynamic torque generated by the tail will be given by the integral of tail position with respect to time, resulting in a time lag between tail movement and body movement.

However, despite the presence of aerodynamic effects, the dominant effect of the tail appears to be inertial. Collecting tail motions in the yaw plane (seven tail swings across five trials) (Fig. 1D, E), we can show correlation between tail angle change and the body angle change during the same time period as the tail motion (*R*^2^ = 0.57, *n* = 7). However, the correlation is not complete, likely a consequence of aerodynamic rotations from feet and tail motion, superimposed upon the inertial effect. While geckos appeared to steer toward the stimulus at the wind tunnel wall, they were not always able to successfully reach it. This could be attributed to the fact that the visual environment does not reflect the optical flow that would be experienced during a true arboreal glide, and more ecologically relevant testing would be needed to assess the aerodynamic authority of *H. platyurus*.

### Planar tail motion modeling

The consequences of scaling the tail up and down in length for a planar yaw are shown in Fig. 3C, D. We see that the angle moved by the body increases as the tail length increases, as would be expected, but when looking at the maneuver speed, calculated as the total angle moved by the body divided by the time taken, we see diminishing returns with increasing tail length. Since inertial reorientation is used for rapid movements with little available time (e.g., falling), the decreasing maneuverability improvement with increasing length will limit the pressure for longer tails. In fact, a maximum maneuver speed is found with a tail that is five times body length for the model parameters used here. 5:1 is a tail length ratio that can be observed in nature (e.g., *Takydromus*), but at such large tail lengths, the rigid body assumptions of the model are not valid, and here we only consider tail lengths up to 3:1, which is more representative of tail size among arboreal lizards.

### Tail trajectory search

Without reaction forces, the final output of the tail trajectory search was a “figure-eight” tail motion, with the tail having to trace opposing motions in pitch and roll to ensure only a pure yaw. This involved repeated changes in tail direction that did not resemble any natural motion observed in the gliding geckos (see supplementary movie). When the reaction forces were added, the tail motion was simpler, and the yawing could be produced in a similar fashion to the cyclic yaw motions observed in the wind tunnel (Fig. 4C). While the trajectory search is somewhat prescriptive (for example, the cost function could take many forms), the difference between the simulations illustrates the importance of aerodynamic reactions to the tail maneuvers seen in the gliding gecko; aerodynamic forces simplify the tail trajectory in certain situations, allowing quick inertial movements in one axis, followed by dissipative recovery strokes using aerodynamic reaction forces.

### Robot data

To test the aerodynamic impact of tail deflection, drop tests were performed with the tail held static deflected upward to 45° and with the tail held parallel to the body (Fig. 5C, E). These tests showed that upward tail deflection produced a constant pitch up change in angle of attack as the robot descended (202.9 ± 10.9°/s, *n* = 6, mean ± SE) while a flat tail posture produced a pitch down at (51.7 ± 3.4°/s, *n* = 6, mean ± SE). Plotting velocity showed that the robot was slowly accelerating, with velocity changing by a mean of 1.8 m/s over the course of a glide (Fig. 5D). The change in posture did not result in a significant change in mean velocity (*P* = 0.38, paired two-tailed *t*-test).

We also tested actuating the tail in mid-flight, with a rapid up and down motion of the tail commanded, switching between the two positions tested statically. These trials showed the expected inertial response of the body, with the body pitching up at the same moment the tail was swung (4° and 6° body pitch). The raised tail then produced a slower pitch up (53 and 27°/s), generated by the aerodynamic forces observed in the static trials. Taken together, the robots trials demonstrate that while inertial forces are dominant, aerodynamic reactions also exert a strong influence on the path of a gliding gecko.

Pressure acts on maneuver speed in several ecological scenarios including signaling mates or conspecifics (e.g., Fleishman and Pallus 2010) for predator evasion Combes and Dudley (2009), Moore et al. (2017) or prey capture Adams and Gifford (2020), and traversing complex habitats (e.g., Higham et al. 2001). The large tails of lizards aid in reorientation during locomotion over complex terrain (Libby et al. 2012) or while airborne (Jusufi et al. 2010). Here, we show that longer tails result in more rapid reorientation (Fig. 3). For example, *H. platyurus* has a tail length to body length ratio of 0.93 and *Anolis carolinensis* has twice the relative tail length ratio of 1.80 (Jusufi et al. 2010, 2011), which results in smaller tail flicks sufficing for reorientation during free fall, and greater effectiveness for turning during gliding. Our modeling results show that this results in a 50% increase in turning rate (60°/s in *H. platyurus* compared to 90°/s in *A. carolinensis*). It should be noted that as tails get even longer, the benefit of a longer tail in reorientation rate diminishes rapidly above a tail length ratio of 2. This relationship between tail length and turning rate poses another challenge for animals with autotomized tails (self-amputated/shed, typically for self-defense). If much of the tail is lost, geckos lose the ability to successfully complete righting after falls (Jusufi et al. 2008). Further, the ability to reorient the body to control a glide trajectory will also be reduced. For example, if a gecko tail is autotomized to 40% of its original length, the turning rate is halved (Fig. 3).

In squamate locomotion, lateral tail oscillation is a common occurrence in the context of back bending that is used in running, climbing, and swimming (Nirody et al. 2018). While we do observe similar planar tail oscillations during aerial behavior (Fig. 1) the observed tail kinematics often have more out of plane motion (see supplementary movie) than during lateral undulation in terrestrial locomotion. During aerial motion, if no external force is applied to the system, then there must be conservation of angular momentum. Thus, if the tail moves to the left and the body responds, when the tail is moved back to the center, the body will in turn respond in the opposite direction, resulting in no net reorientation. The observed out of plane motions of the tail, allow the tail to continue rotating in one direction to allow a maneuver to occur over multiple sweeps of the tail.

Our analyses also found a significant but not dominant aerodynamic role for the tail. It is important to note here that our experiments examine the gecko at or near to terminal velocity, whereas previous observations of the air righting reflex in geckos have been made at the onset of a fall, that is, where velocity is zero. The importance of terminal velocity falling to the animal’s life cycle is difficult to quantify, but we can gain insight into the relevance of terminal speed by calculating the distance the animal must fall to reach terminal velocity (see Appendix 2 for full calculation). Based on the typical glide speed (6 m/s), mass (2.9 g), and approximate projected area (890 mm^2^) of *H. platyurus*, we can estimate its drag coefficient as 1.9 (this drag coefficient is significantly higher than a flat plate of equivalent area, reflecting some aerodynamic adaptation). Based on that drag coefficient, the distance a falling gecko must descend vertically to reach 75% of terminal speed can be estimated as 1.6 m. To reach 95% terminal speed, the gecko must descend 4.1 m. These are feasible falling heights for wild geckos, given the height of the canopy they inhabit.

Falling at terminal velocity changes the dynamics of reorientation significantly, and as terminal velocity changes with size (Haldane 1926), we can also expect the relative contribution from aerodynamic and inertial effects to change significantly. The available torque that can be applied to the body in an inertial reorientation is simply the torque that can be applied to the inertial appendage, which in this instance would be proportional to the muscle cross-section (*length*^2^) multiplied by a lever arm length, giving an overall scaling of *length*^3^. An aerodynamic torque depends on appendage area, lever arm length and velocity, that is, *length*^3^ multiplied by *velocity*^2^. At terminal velocity, *velocity*^2^ is proportional to mass divided by area, or proportional to *length*^1^ (where mass scaling is isometric). This would mean that the available aerodynamic torque is proportional to *length*^4^. The faster scaling up of aerodynamic torque relative to inertial torque as size increases may partially explain why a larger arboreal mammal such as *Sciurus carolinensi* (Fukushima et al. 2021) exhibits a smaller relative tail mass (3% body mass, vs. 10% in *H. platyurus*), but has adaptations to increase aerodynamic area through fur growth (tail fur produces a significant increase in aerodynamic area; Patel et al. 2016).

The effects of inertial forces at low speeds during righting (e.g., Jusufi et al. 2008; Jusufi et al. 2010; Jusufi et al. 20111), leaping (e.g., Dunbar 1988), or running Libby et al. (2012) are well described. Similarly, the aerodynamic torques used by falling or gliding animals to reorient using an outstretched limb or tail are well established from wind-tunnel studies (McCay 2001; Stein et al. 2008; Munk et al. 2015). By examining descent at higher speeds our data show that both inertial and aerodynamic forces interact to affect reorientation and their relative contributions vary with increasing speed. This interaction between inertial and aerodynamic forces needs to be further explored, particularly in animals that do not appear specialized for gliding. The robot and code presented in this paper both use rigid tails, but advances in soft robotics (Jusufi et al. 2017; Wolf et al. 2020; Lin et al. 2021; Siddall et al. 2021) mean that testing the effect of lifelike, compliant tails with many degrees of freedom will increasingly become feasible in future studies to enable soft robotic physical models with near presensile dexterous capabilities. Numerous arboreal animals are at risk of falling and once airborne this interaction of forces plays a role in their ability to reorient and land safely.

## Conclusion

Lizard tails vary widely in form and function, shaped by their role in signaling, camouflage, and locomotion. Tail proportions are often associated with habitat and those differences can have an influence on the mechanics of locomotion. In arboreal lizards, that risk falls from height, tail proportions affect an animal’s ability to reorient in the air and control trajectory to land safely. For tree-dwelling lizards, relatively longer tails potentially allows for more rapid reorientation and reduced control effort required for of attaining a desired body orientation through use of both inertial and aerodynamic forces. Coordinated responses with respect to these forces appear required to successfully control posture in the air. These data only reinforce the versatility of tails and their importance in the evolutionary success of lizards and other tailed animals.

In this paper, we have examined the role of tails in gliding locomotion with several new biomechanical and biorobotics experiments complemented by analytical models. Results suggest that multiple coordinated tail responses are necessary for diverse model systems to control body posture during jumping, mid-air righting and directed aerial descent. Future work will combine the trajectory optimization shown here with more developed physical models in subsequent versions of the robot platform presented here, to test tail use in multiple degrees of freedom across a range of glide conditions.

## Supplementary Material

icab132_Supplemental_File

## Figures and Tables

**Fig. 6:**
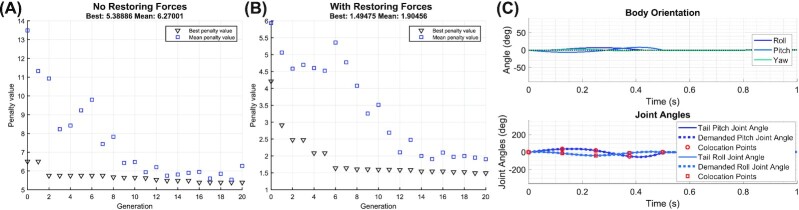
(**A**) Trajectory optimization progression for the result displayed in Fig. 4D, without restoring forces. (**B**) Trajectory optimization progression for the result displayed in Fig. 4E, with restoring forces. (**C**) Example of trajectory optimization result with no seeding, and no external forces included. The optimization finds a local minimum with no net body motion. To avoid this, 50% initial population seeding was added with a coarse representation of the observed gecko tail motion.

## Data Availability

A video file is included with the paper (Title image in video attachment is copyright 2008 National Academy of Sciences). The model code can be found here: dx.doi.org/10.17617/3.6k or here: github.com/robjds/Tailobatics. The other data presented in this paper will be made available upon reasonable request.

## Appendix 1: Trajectory optimization detail

The Matlab code for both the multibody simulink model and the optimization procedure is available for download, see the “Data availability” section. The demanded tail trajectory is input to the simulation as a cubic spline between three collocation points at fixed points in time (Fig. 4D, E). The resultant tail motion in each axis is determined by a PID position controller, where the process variable is the angle of each tail joint and the setpoint is the demanded tail value at a given instant in time. The controller determines the instantaneous torque applied to each tail axis, and the same PID gains are used on both axes. These gains are listed in Table 1.

**Table 1:** Specifications and variable definitions of the multibody model

Parameter	Value
Body mass	2.9 g, Jusufi et al. (2010)
Body length	54.0 mm, Jusufi et al. (2010)
Tail mass	0.29 g, Jusufi et al. (2010)
Tail length	50.0 mm, Jusufi et al. (2010)
External rate, *C*_1_	5 × 10^−7^ Nm/°
External damping, *C*_2_	0.5 × 10^−7^ Nm/°/s
*Tail axis gains:*	
Proportional gain	0.1 Nm/rad
Integral gain	0.05 Nm/rad/s
Derivative gain	0.02 Nm s/rad

The trajectory optimization is run using the inbuilt genetic algorithm function in Matlab (2020). We ran the optimization using default settings, with a fixed “population” and number of “generations,” in this case 20 and 20, respectively, requiring 400 simulations of the model, with the best value after 20 generations taken as the result (Fig. 6). The objective function is made up of a path length constraint (found by integrating the motion of the tail pitch joint θ and roll join, ψ), the absolute difference between the final body angle and the demanded body angle change (which in this case was 0° in pitch, α, 0° in roll, β, and 30° in yaw, γ), and a body velocity penalty such that the cost function, *F*_cost_ was

(3) $$\begin{eqnarray*}
F_{\mathrm{ cost}} = F_{\mathrm{ angle}} + F_{\mathrm{ velocity}} + F_{\mathrm{ path}}
\end{eqnarray*}$$

(4) $$\begin{eqnarray*}
F_{\mathrm{ angle}} = |\alpha | + |\beta | + ||\gamma |-\frac{\pi }{6}|
\end{eqnarray*}$$

(5) $$\begin{eqnarray*}
F_{\mathrm{ velocity}} = |\dot{\alpha }| + |\dot{\beta }| + |\dot{\gamma }|
\end{eqnarray*}$$

(6) $$\begin{eqnarray*}
F_{\mathrm{ path}} = \int |\theta | + |\psi | \mathrm{ d}t
\end{eqnarray*}$$

To limit the search space, half of the population of initial guess collocation points (i.e., 10 tail trajectories out of 20 in the population) are prescribed as [90°, 90°, 90°] in tail pitch and [90°, 0°, −90°] in tail roll, with the other 10 guesses generated randomly within the possible tail motion range (±100° in tail pitch and ±370° in tail roll). The 10 prescribed guesses represent a coarse representation of the observed gecko tail motion (see supplementary video), to focus the trajectory search. Removing this seeding and beginning the trajectory search with entirely random collocation points resulted in a local minima with a net body yaw motion of 0.5° vs. 5.5° yaw produced by seeding the optimization with approximate animal motion (Fig. 6C). The remaining parameters for the model are tabulated in Table 1, including the external torque constants *C*_1_ and *C*_2_ [Equations (1) and (2)]. Body mass and length parameters are taken from Jusufi et al. (2010).

## Appendix 2: Terminal velocity fall distance

For an object falling under quadratic drag, terminal velocity will be reached when drag balances body weight, such that

(7) $$\begin{equation*}
\frac{1}{2} \rho A C_\mathrm{ D} v_\mathrm{ T}^2 = mg
\end{equation*}$$

where *m* is mass, *A* is area, *C*_D_ is drag coefficient, *g* is acceleration due to gravity, and ρ is air density. The terminal velocity is then

(8) $$\begin{equation*}
v_\mathrm{ T} = \left( \frac{2mg}{\rho AC_\mathrm{ D}} \right)^{\frac{1}{2}}
\end{equation*}$$

For an object descending vertically in a straight line from rest, the instantaneous velocity, *v*, at time *t* is

(9) $$\begin{equation*}
v = v_\mathrm{ T} \tanh {\left(\frac{tg}{v_\mathrm{ T}}\right)}
\end{equation*}$$

which can be integrated to give distance, *x* as a function of time:

(10) $$\begin{equation*}
x = \frac{{v_\mathrm{ T}}^2}{g}\ln {\left(\cosh {\left(\frac{tg}{v_\mathrm{ T}}\right)}\right)}
\end{equation*}$$

These equations can be used to find the distance traveled before a falling object reaches a significant proportion of its terminal velocity.
